# All-Inorganic Perovskite Solar Cells Based on CsPbIBr_2_ and Metal Oxide Transport Layers with Improved Stability

**DOI:** 10.3390/nano9121666

**Published:** 2019-11-22

**Authors:** Jien Yang, Qiong Zhang, Jinjin Xu, Hairui Liu, Ruiping Qin, Haifa Zhai, Songhua Chen, Mingjian Yuan

**Affiliations:** 1 Henan Key Laboratory of Photovoltaic Materials, College of Physics and Materials Science, Henan Normal University, Xinxiang 453007, China; qiongzhang01@163.com (Q.Z.); jinjinxuhtu@163.com (J.X.); Liuhairui1@126.com (H.L.); qinruiping@163.com (R.Q.); zhaihaifa@htu.edu.cn (H.Z.); 2 College of Chemistry and Material Science, Longyan University, Longyan 364012, China; 3 College of Chemistry, Nankai University, Tianjin 300071, China; yuanmj@nankai.edu.cn

**Keywords:** inorganic perovskite, CsPbIBr_2_, metal oxide transport layers

## Abstract

Despite the successful improvement in the power conversion efficiency (PCE) of perovskite solar cells (PSCs), the issue of instability is still a serious challenge for their commercial application. The issue of the PSCs mainly originates from the decomposition of the organic–inorganic hybrid perovskite materials, which will degrade upon humidity and suffer from the thermal environment. In addition, the charge transport layers also influence the stability of the whole devices. In this study, inorganic transport layers are utilized in an inverted structure of PSCs employing CsPbIBr_2_ as light absorbent layer, in which nickel oxide (NiO_x_) and cerium oxide (CeO_x_) films are applied as the hole transport layer (HTL) and the electron transport layer (ETL), respectively. The inorganic transport layers are expected to protect the CsPbIBr_2_ film from the contact of moisture and react with the metal electrode, thus preventing degradation. The PSC with all inorganic components, inorganic perovskite and inorganic transport layers demonstrates an initial PCE of 5.60% and retains 5.56% after 600 s in ambient air at maximum power point tracking.

## 1. Introduction

The solar cells adopting organic–inorganic hybrid perovskite as absorber have obtained significant attention in the field of photovoltaics, with an unprecedented rise in power conversion efficiency (PCE) from 3.8% to 25.2% in a few years [1]. Although the highest certified efficiency of the PSCs is comparable to that of commercialized silicon photovoltaic devices, the issue of instability of PSCs has prevented its practical application. In particular, the volatile and hygroscopic nature of the organic cations such as methyl ammonium and formamidinium makes the organic–inorganic hybrid perovskite materials become more unstable under illumination, thermal, moisture and stresses [2,3].

In recent years, cesium lead halide or mixed halide perovskites CsPbX_3_ have attracted much attention as light absorbent layer materials due to their excellent thermal stability as well as suitable photophysical properties [4,5,6]. In particular, the CsPbIBr_2_ have emerged as a better candidate among its cousins, because of its capacity to balance the bandgap and its thermal stability [7], which has a bandgap of 2.05 eV, promising a potential highest PCE about 17.5% according to the Shockley–Queisser limit [8] and the material is unexpectedly stable with a melting point more than 460 °C under ambient atmosphere [9]. The higher performance PSCs usually have a sandwich structure, in which the perovskite active layer is placed between a hole transport layer (HTL) and an electron transport layer (ETL). The transport layers not only promote the transportation of the photogenerated carriers produced in the perovskite active layer and prevent carrier recombination at the interfaces, but also form ohmic contact with the electrode. Typically, in a n-i-p structure, the perovskite materials usually are deposited on a compact TiO_2_ layer and an optional mesoporous TiO_2_ scaffold layer [10], and covered with a layer of doping 2,2′,7,7′-tetrakis(*N,N*-*p*-dimethoxyphenylamino)-9,9′-spirobifluorene (Spiro-OMeTAD) sequentially. As a counterpart, the p–i–n structure, perovskite materials are deposited onto the film of poly(3,4-ethylene dioxythiophene):polystyrene sulfonic acid (PEDOT:PSS) or poly[bis(4-phenyl)(2,4,6-trimethylphenyl)amine] (PTAA ) and then covered with a layer of [6,6]-phenyl-C61-butyric acid methyl ester (PCBM) or C_60_. However, PCBM and Spiro-OMeTAD are expensive and the doping agents of Spiro-OMeTAD could break down the electrode and perovskite layers gradually. To achieve novel PSCs with long-term stability and low-cost of fabrication, the strategies of replacing the organic transport layers with inorganic materials such as CuSCN [11], CuI [12], NiO_x_ [13] as the hole transport layers and ZnO [14], CdS [15], SnO_2_ [16] as the electron transport layers has been extensively explored. The inorganic metal oxides have much higher carrier mobility and superior stability as well as easy-processing from corresponding precursors. Herein, we use cerium oxide (CeO_x_) as electron transport layers with in situ decomposition from its precursor at low temperature (100 °C) and nickel oxide (NiO_x_) as hole transport layers from pre-prepared NiO_x_ nanoparticles solution as well as CsPbIBr_2_ perovskite as the photoactive layer in the device. The prepared perovskite solar cells with a configuration of ITO/NiO_x_/CsPbIBr_2_/CeO_x_/Ag based on an all-metal-oxide charge transport layer and the all-inorganic perovskite material shows 5.60% efficiency and significantly improved stability compared to those reported [17,18].

## 2. Materials and Methods 

### 2.1. Materials

Sodium hydroxide, Chlorobenzene, Cerium (III) acetylacetonate hydrate and Ni(NO_3_)_2_·6H_2_O were purchased from Aladdin. Reagent (Shanghai, China) Co., Ltd. Cesium iodide and lead bromide were purchased from Xinxiang Aode New Mater. Co., Let (Xinxiang, China). AGC ITO was purchased from Youxuan Co., Let (Yingkou, China) with 1.1 mm, 7~9 Ω/sq and transmittance about 88%. Dimethyl sulfoxide (DMSO) was purchased from Sigma-Aldrich. All chemicals were used without purification.

### 2.2. Methods of Nanoparticles Preparation and Device Fabrication

#### 2.2.1. Preparation of NiO_x_ Nanoparticles

The NiO_x_ nanoparticles were prepared according reported lecture with a little modification [19]. To be specific, A clear green solution of nickel (II) salt was obtained by dissolving 14.55 g (0.05 mol) Ni(NO_3_)_2_·6H_2_O in 100 mL deionized water with vigorous stirring. Then a NaOH aqueous solution of 10 mol L^−1^ was added to the solution until the pH value reached 10. After another 10 min’ stirring, the obtained green precipitants through suction filtration were transferred to vacuum oven and dried 6 hours at 80 °C under reduced pressure. The NiO_x_ nanoparticles were prepared as dark gray powders by calcining the dried precipitants at 270 °C for 2 h.

#### 2.2.2. Preparation of Precursor Solutions of CeOx and CsPbIBr_2_

The cerium oxide precursor solution was obtained by dissolving cerium (III) acetylacetonate hydrate (44 mg, 0.1 mmol) in 10 mL chlorobenzene after 30 min treatment of ultrasonication [20,21].

The perovskite (CsPbIBr_2_) precursor solution was prepared by mixing 2.34 g cesium iodide (CsI) and 3.30 g lead bromide (PbBr_2_) with equal molar ratio in 10 mL dimethyl sulfoxide (DMSO) with stirring at 60 °C until all the solid dissolved completely.

#### 2.2.3. Device Fabrication

The pre-patterned indium tin oxide (ITO) glass was cleaned in ultrasonic sequential with detergent, deionized water, acetone, and isopropanol for 15 min, dried in N_2_, followed by treatment of plasma for 10 min. The film of NiO_x_ was deposited on the ITO substrate using 0.5 mg mL^−1^ NiO_x_ nanoparticles aqueous solution by spin coating at 2500 r.p.m. for 60 s, and annealed at 150 C for 30 min in air. Then, the perovskite precursor solution was spin-coated on the NiO_x_ film at 2500 r.p.m. for 53 s and annealed on a hot plate at 100 °C for 20 min. The ETL of CeO_x_ was fabricated on the top of the perovskite layer by spin-coating the precursor at 2000 r.p.m. for 30 s and annealed in N_2_ at 100 °C for 10 min. Finally, 100 nm Ag was thermally evaporated on the ETL as the top metal electrode under a pressure less than 5 × 10^−4^ Pa.

### 2.3. Characterization

The top-view and cross-section morphologies of perovskite films of PSC was characterized using a field-emission scanning electron microscope (FESEM, Quanta 200 FEG, FEI Co.). Ultraviolet photoelectron spectroscopy (UPS) measurements were measured on a ThermoFisher ESCALAB 250Xi instrument using the HeI (21.22 eV) emission line. Water contact angle on these films were recorded with JC000DI contact angle measuring instrument (Zhong Chen. Shanghai, China). Atomic force microscopy (AFM) images were obtained using a Veeco MultimodeV instrument to evaluate the surface morphology of films in the tapping and intelligent mode. The steady state photoluminescence spectra (PL) and time-resolved photoluminescence spectra (TRPL) were acquired on an FS5 spectrometer from Edinburgh Instruments excited with 500 W Xenon lamp and 5 mW picoseconds pulsed diode laser at 410 ± 10 nm. The solar cells were measured using simulated air mass 1.5 global sunlight (AM 1.5G) conditions (100 mW cm^−2^) with 2400 Source Meter, Keithley Instruments. The external quantum efficiency (EQE) of the solar cells were measured using a combined system, including a xenon lamp, monochromator, chopper and lock-in amplifier together with a calibrated silicon photodetector. Perovskite films’ ultraviolet–visible (UV–Vis) absorption measurements were performed through a Shimadzu UV-3600 spectrometer.

## 3. Results

**Figure 1:**
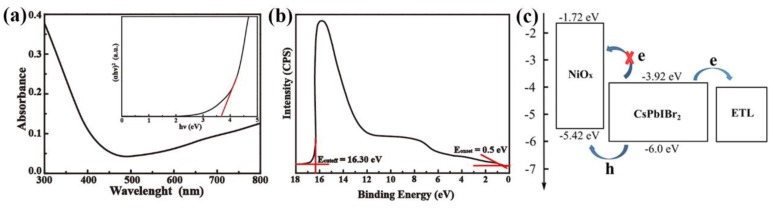
(**a**) Ultraviolet–visible (UV-Vis) absorption spectra of NiO_x_ film deposited on glass substrate. (The inset is the plots of the (αhν)^2^ versus energy). (**b**) Ultraviolet photoelectron spectroscopy (UPS) spectra of the NiO_x_ film deposited on the ITO substrate. (**c**) The band alignment between a NiO_x_ film and perovskite film.

NiO_x_ was applied in this work as HTL. The optical and electric properties were measured with UV-Vis spectra and ultraviolet photoelectron spectroscopy (UPS) (Figure 1).

As shown in Figure 1a, the bandgap of NiO_x_ film could be evaluated from the UV–Vis absorbance spectrometry. The direct bandgap energy (*E_g_*) can be determined using the direct transition equation [22] ${\left(\alpha h\nu \right)}^{2}=A\left(h\nu -E_{g}\right)$. The plot of (*αhv*)^2^ versus *hv* is given in the inset of Figure 1a, and the curve is obtained by the M-K equation. *E_g_* for the NiO_x_ film is determined to be 3.7 eV. The UPS of the NiO_x_ film is shown in Figure 1b, indicating the work function of the film. The calculated results of the valence band energy level as shown in Figure 1c is 5.42 eV, facilitating the extraction of holes from perovskite, while the conductive band energy level could be calculated as 1.72 eV through the optical bandgap and the valence band energy level, which is higher than that of CsPbIBr_2_ as shown in Figure 1c, blocking the injection of electrons. 

**Figure 2:**
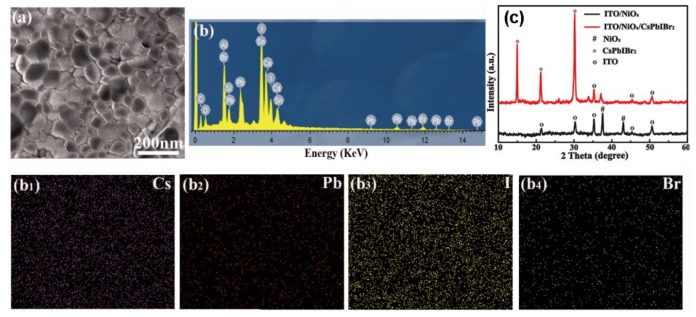
(**a**) Top view scanning electron micrograph (SEM) of CsPbIBr_2_ film. (**b**) The EDS spectra. (**c**) X-ray diffraction (XRD) of ITO/NiO_x_ and ITO/NiO_x_/CsPbIBr_2_. (**b_1–4_**) The EDS elemental mappings of Cs, Pb, I and Br throughout CsPbIBr_2_ film.

Generally, the morphology of perovskite film can influence the performance of perovskite solar cells. The morphology of the CsPbIBr_2_ films is characterized by the scanning electron microscopy (SEM) images (Figure 2). The image of the top-view SEM of CsPbIBr_2_ film on the top of the NiO_x_ in Figure 2a exhibits a compact and smooth film on the top of the NiO_x_ without any pin holes. Furthermore, the energy dispersive spectrometer EDS mapping of corresponding element Cs, Pb, I and Br are homogeneously distributed throughout the perovskite film without phase separation shown in Figure 2b, confirming the full coverage of CsPbIBr_2_ film on the NiO_x_ film, partially guaranteeing the high performance of the perovskite solar cells. X-ray diffraction (XRD) measurements were carried out to reveal the formation of CsPbIBr_2_ perovskite (Figure 2c). The diffraction peaks in the XRD patterns were consistent with those of [23]. 

**Figure 3:**
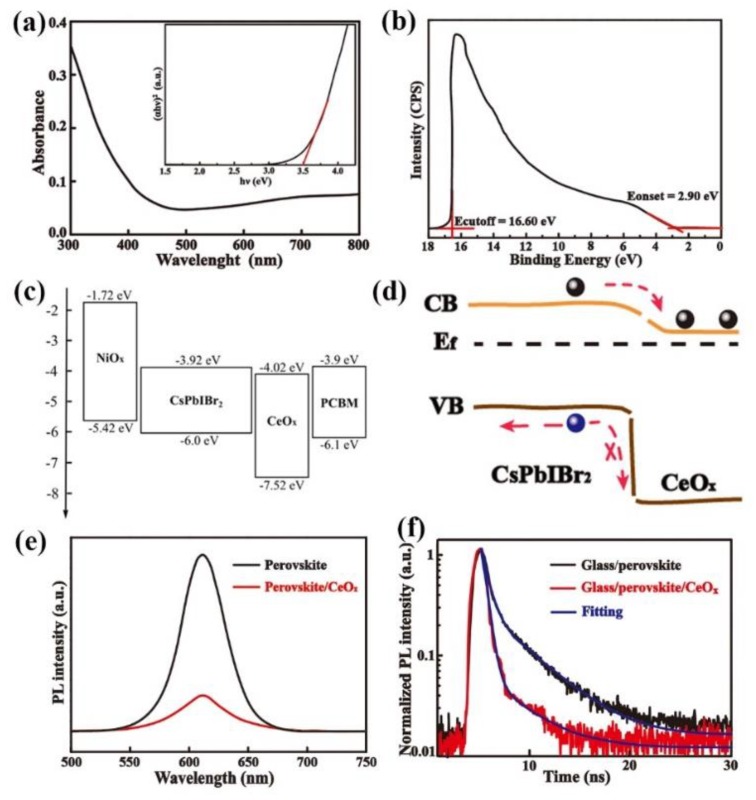
(**a**) UV-vis absorption spectra of CeO_x_ film. (The inset is plots of the (αhν)^2^ versus energy). (**b**) UPS spectra of the CeOx film. (**c**) The energy band diagram of the components in the device. (**d**) the diagram of energy bands bends downwards at the CsPbIBr_2_/CeO_x_ interface. (**e**) The steady-state photoluminescence (PL) spectra. (**f**) The time-resolved PL spectra.

The performance of device depends not only on the morphology of the film but also on the separation of exciton and the charge carrier’s mobility through the interfaces. The charge transport layers widely are employed to enhance carrier injection, decrease trap-states, and reduce contact resistance [24]. In this work, another inorganic transport material, CeO_x_ is used as the electron transport layer (ETL) in inverted CsPbIBr_2_ PSCs. In order to research how the energy level align, we measured the bandgap of the CeO_x_ layer. The *E_g_* of CeO_x_ film could be obtained from the UV-Vis absorbance spectrometry in Figure 3a, and which is determined to be 3.5 eV. The UPS of the CeO_x_ film is shown in Figure 3b, and the valence band (*E_VB_*) of the CeO_x_ film is calculated to be 7.52 eV. According to the relationship of $E_{\mathrm{CB}}=E_{\mathrm{VB}}+E_{g}$, the conductive band energy level (*E_CB_*) of CeO_x_ is calculated as 4.02 eV. Compared to the conventional ETL (PCBM), CeO_x_ shows a better energy level match with CsPbIBr_2_ due to it has a lower valence band energy level, the larger driving force will facilitate the election injection and blocks the holes. At the same time, the CeO_x_ energy level of the conductive band is slightly lower than CsPbIBr_2_, proving CeO_x_ can be used as a suitable ETL for CsPbIBr_2_ PSCs (Figure 3c).

The conductive band energy level of CsPbIBr_2_ and CeO_x_ discontinuity (Δ*E_c_*) is 0.1 eV, the valence band energy level discontinuity is 1.52 eV, which means CeO_x_ layer can actually be an electronic transfer tunnel when the energy bands bend downwards, decreasing the energy barrier from the conductive band of perovskite absorber (Figure 3d). To further demonstrate the electron extraction mechanism, the steady-state photoluminescence (PL) and TRPL spectra were measured and are shown in Figure 3e,f This shows that the CsPbIBr_2_/CeO_x_ exhibit more efficient PL quenching ability, the significantly lower PL intensity indicates a stronger interfacial electron extraction efficiency from perovskite absorber layer [25,26]. The two TRPL spectra are fitted with a bi-exponential decay function [27], and the PL decay time of glass/CsPbIBr_2_ are τ_1_ = 0.53 ns, τ_2_ = 3.07 ns. While that of glass/CsPbIBr_2_/CeO_x_ are τ_1_ = 0.38 ns, τ_2_ = 2.58 ns. The reduction of the PL decay time indicates a more efficient electron extraction capability. Furthermore, the presence of CeO_x_ can speed up the electron extract and increase the photovoltaic performance of the inorganic PSCs.

**Figure 4:**
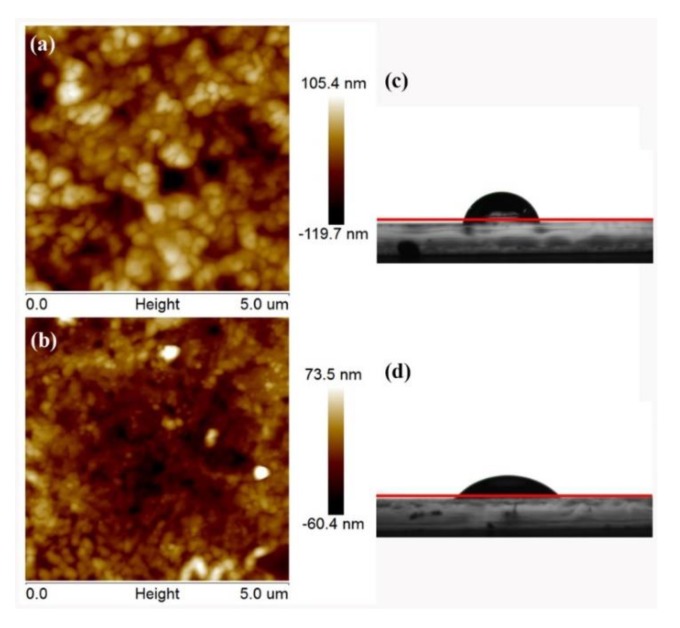
Two-dimensional (2D) tapping-mode atomic force microscopy (AFM) height (**a**) ITO/CsPbIBr_2_. (**b**) ITO/CsPbIBr_2_/CeO_x_. The water contact angle: (**c**) ITO/CsPbIBr_2_. (**d**) ITO /CsPbIBr_2_/CeO_x_.

The contact resistance strongly depends on adhesion of contact interface and the flatness of the contact interface. To estimate the roughness of the contact interface with and without depositing CeO_x_ film, the AFM measurements were tested (Figure 4a,b). The root-mean-square (RMS) roughness of the pure perovskite was 19.17 nm, the RMS roughness of the CsPbIBr_2_/CeO_x_ film was 11.86 nm, the reduced RMS roughness would be beneficial in decreasing the contact resistance after deposited CeO_x_ layer. The water contact angle on these films is also tested (Figure 4c). The contact angle is 63.1° for the pure perovskite film, while 34.8° for the perovskite/CeO_x_ film. A relatively good wetting property can have a better atomic adsorption compared to the non-wetting surfaces, it can also contribute to low electrical contact resistance [28].

**Figure 5:**
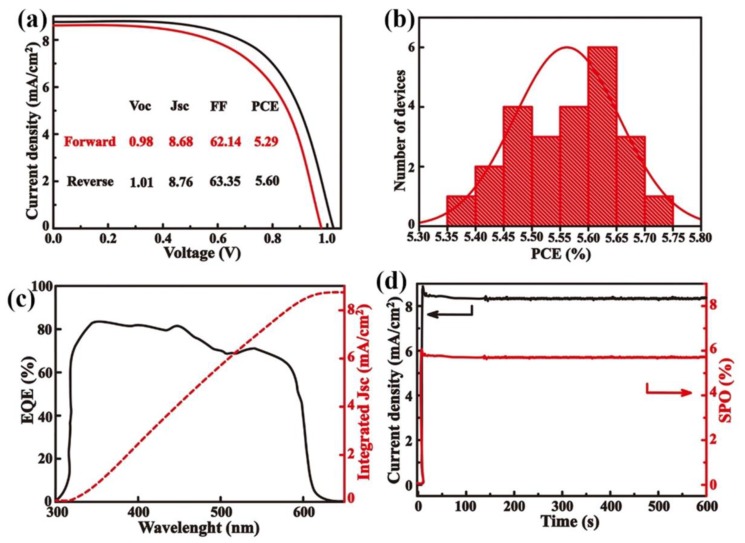
(**a**) The current-voltage (*J-V)* curves of perovskite solar cells measured in different scan directions. (**b**) Statistical power conversion efficiencies (PCEs) of 24 independent cells. (**c**) The corresponding external quantum efficiency (EQE) spectrum together with the integrated *J_sc_* of the champion CsPbIBr_2_ PSC. (**d**) Steady-state maximum PCE outputs.

To evaluate the influence of the metal oxide transport layers on the device performance, an inverted planar CsPbIBr_2_ PSCs with a configuration of ITO/NiO_x_/CsPbIBr_2_/CeO_x_/Ag were fabricated. The current-voltage (*J-V*) curve of the inorganic PSCs is shown in Figure 5a. The best power conversion efficiency (PCE) of 5.60% was obtained in the device with an open-circuit (*V_oc_*) of 1.01 V, a short-circuit current density (*J_sc_*) of 8.76 mA cm^−2^, a fill factor (FF) of 63.35%. The device also shows a small *J-V* hysteresis (Figure 5a). Figure 5b gives statistical PCEs of 24 independent cells. Average PCE is calculated to be 5.56% for all the inorganic PSCs. 

The external quantum efficiency (EQE) spectrum of the device was measured and is shown in Figure 5c. The corresponding integrated *J_sc_* of 8.71 mA cm^−2^, which is close to the *J_sc_* derived from the *J-V* curve. This result indicates that the device has a better photoelectric conversion property. The stabilized power output (SPO) of the PSCs is also shown in Figure 5d, and the stabilized efficiency output is estimated to 5.54% for the solar cell after being measured for over 600 s. In addition, the PCE output is much more stable during long time measurement, indicating the inorganic materials can have a better stability. 

**Figure 6:**
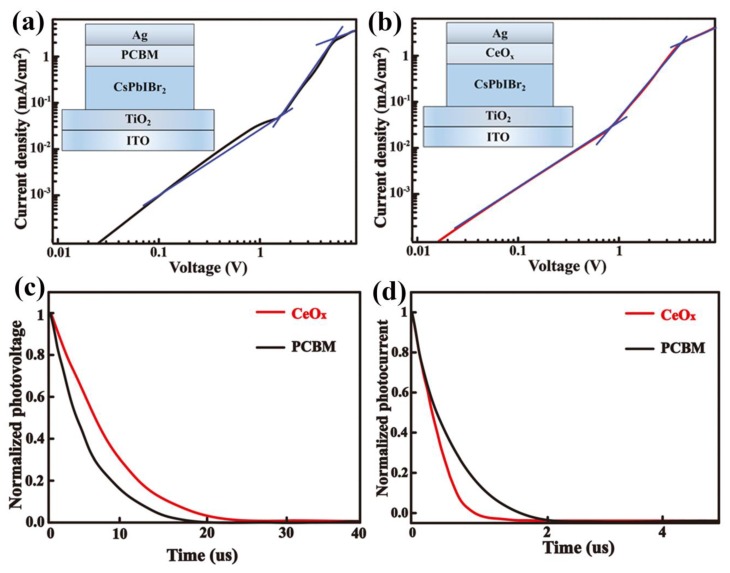
(**a**) The space-charge-limited current (SCLC) plot of ITO/ TiO_2_/perovskite/[6,6]-phenyl-C61-butyric acid methyl ester (PCBM) /Ag devices. (**b**) The SCLC plot of ITO/ TiO_2_/perovskite/CeO_x/_Ag devices. (**c**) The transient photovoltage (TPV) decay curves. (**d**) The transient photocurrent (TPC) decay curves.

The electrical property of these inorganic materials is investigated to determine they are better adapted to organic materials used in the PSCs. The space-charge-limited current (SCLC) measurement has been tested. We measured the electron-only devices and these are shown in the Figure 6a,b. The SCLC can be divided into three sections: ohmic regime, trap-filling regime and trap-free SCLC regime. The trap-state density (*N_trap_*) can be determined by the trap-filled limit voltage equation [29,30], and the *N_trap_* of the PCBM and CeO_x_ ETL are 6.95 × 10^16^ and 3.89 × 10^16^ cm^−3^, respectively, indicating the CeO_x_ as the ETL can reduce the trap density. The mobility of the devices is calculated using Equation [31] $J=9\varepsilon_{0}\varepsilon_{r}\mu V^{2}/8L^{2}$, the mobility values of these devices are 0.39 cm^2^ V^−1^ S^−1^ and 1.26 cm^2^ V^−1^ S^−1^, respectively. This result implies the device with the CeO_x_ exhibits more efficient electron extraction from the perovskite layer. 

To further investigate the carrier transfer and recombination in the inorganic PSC, the transient photovoltage (TPV) and transient photocurrent (TPC) measurements are carried out. Figure 6d shows the photocurrent decay of the different ETL devices measured at short current condition. The CeO_x_-based device has faster decay than the PCBM-based device, suggesting that the CeO_x_-based device has a better carrier extraction and transport. The TPV is carried out to measure the charge recombination lifetime (Figure 6c). The devices with CeO_x_ ETL demonstrates a slower photovoltage decay, indicating the charge recombination can be suppressed and lower trap density. This situation is consistent with the PL and SCLC results that improve the charge transfer and decrease the trap states in the all inorganic PSCs.

**Figure 7:**
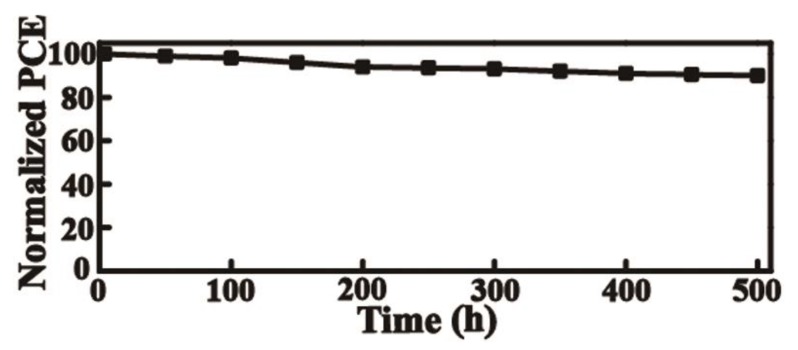
The normalized efficiency decay of perovskite solar cells.

Finally, the stabilities of the devices are examined. All the devices are evaluated and the results are shown in Figure 7. These devices are stored in an atmospheric condition at 40–45 °C and humidity in the 45–50% without encapsulation. 

It can be seen that the PCE of the device with the CeO_x_ ETL can retain 90% of its initial efficiency after 500 h. Generally, the water can damage the perovskite layer and cause decomposition [32]. However, the CeO_x_ film can protect the perovskite layer from water and has more stability in air. According to the previous report, the organic ETL is not dense enough against the water and makes the perovskite layer degenerate. In contract, the inorganic ETL can prevent the perovskite layer from water and act as a diffusion barrier to prevent the metal electrode decomposing. 

## 4. Conclusions

In summary, cost-effective, efficient p-i-n PSCs were demonstrated with an inorganic perovskite layer sandwiched between two metal oxides transport layers. The inorganic transport layers retarded the degradation of the perovskite film and decreased the contact resistance, trap density between the interfaces of the CsPbIBr_2_ film and the Ag electrode effectively. Furthermore, the suitable energy alignments of the layers increased the performance of the PSCs with a *V_oc_* of 1.01 V and *J_sc_* of 8.76 mA cm^−2^. The best PCE of the devices is of 5.60% with a stabilized value of 5.56%. In addition, the high-quality all inorganic PSC show a long-term endurance against the influences of heat and humidity. Therefore, the all inorganic materials involved in PSCs provide a novel approach to fabricate future cheap and stable perovskite solar cells with an expectation of practicability.

## References

[1] NREL Best Research Efficiencies. https://www.nrel.gov/pv/cell-efficiency.html.

[2] Conings B., Drijkoningen J., Gauquelin N., Babayigit A., D’Haen J., D’Olieslaeger L., Ethirajan A., Verbeeck J., Manca J., Mosconi E. (2015). Intrinsic Thermal Instability of Methylammonium Lead Trihalide Perovskite. Adv. Energy Mater..

[3] Yang J., Siempelkamp B.D., Liu D., Kelly T.L. (2015). Investigation of CH3NH3PbI3 Degradation Rates and Mechanisms in Controlled Humidity Environments Using in Situ Techniques. ACS Nano.

[4] Beal R.E., Slotcavage D.J., Leijtens T., Bowring A.R., Belisle R.A., Nguyen W.H., Burkhard G.F., Hoke E.T., McGehee M.D. (2016). Cesium lead halide perovskites with improved stability for tandem solar cells. J. Phys. Chem. Lett..

[5] Kim G.-W., Kang G., Malekshahi Byranvand M., Lee G.-Y., Park T. (2017). Gradated mixed hole transport layer in a perovskite solar cell: Improving moisture stability and efficiency. ACS Appl. Mater. Interfaces.

[6] Protesescu L., Yakunin S., Bodnarchuk M.I., Krieg F., Caputo R., Hendon C.H., Yang R.X., Walsh A., Kovalenko M.V. (2015). Nanocrystals of cesium lead halide perovskites (CsPbX3, X= Cl, Br, and I): Novel optoelectronic materials showing bright emission with wide color gamut. Nano Lett..

[7] Zhang L., Zhang Q., Xing X., Jiang Y., He T., Huang Y., Ma Z., Yang J., Yuan M. (2018). Conjugated Alkylamine by Two-Step Surface Ligand Engineering in CsPbBr3 Perovskite Nanocrystals for Efficient Light-Emitting Diodes. ChemNanoMat.

[8] Shockley W., Queisser H.J. (1961). Detailed Balance Limit of Efficiency of p-n Junction Solar Cells. J. Appl. Phys..

[9] Liu C., Li W., Li H., Wang H., Zhang C., Yang Y., Gao X., Xue Q., Yip H.-L., Fan J. (2019). Structurally Reconstructed CsPbI2Br Perovskite for Highly Stable and Square-Centimeter All-Inorganic Perovskite Solar Cells. Adv. Energy Mater..

[10] Ma J., Chang J., Lin Z., Guo X., Zhou L., Liu Z., Xi H., Chen D., Zhang C., Hao Y. (2018). Elucidating the roles of TiCl4 and PCBM fullerene treatment on TiO2 electron transporting layer for highly efficient planar perovskite solar cells. J. Phys. Chem. C.

[11] Arora N., Dar M.I., Hinderhofer A., Pellet N., Schreiber F., Zakeeruddin S.M., Grätzel M. (2017). Perovskite solar cells with CuSCN hole extraction layers yield stabilized efficiencies greater than 20%. Science.

[12] Gharibzadeh S., Nejand B.A., Moshaii A., Mohammadian N., Alizadeh A.H., Mohammadpour R., Ahmadi V., Alizadeh A. (2016). Two-step physical deposition of a compact CuI Hole-Transport layer and the formation of an interfacial species in perovskite solar cells. ChemSusChem.

[13] Mali S.S., Kim H., Kim H.H., Shim S.E., Hong C.K. (2018). Nanoporous p-type NiOx electrode for pin inverted perovskite solar cell toward air stability. Mater. Today.

[14] Ahmad S., Fu P., Yu S., Yang Q., Liu X., Wang X., Wang X., Guo X., Li C. (2019). Dion-Jacobson Phase 2D Layered Perovskites for Solar Cells with Ultrahigh Stability. Joule.

[15] Hwang I., Yong K. (2016). Novel CdS hole-blocking layer for photostable perovskite solar cells. ACS Appl. Mater. Interfaces.

[16] Jiang Y., Yuan J., Ni Y., Yang J., Wang Y., Jiu T., Yuan M., Chen J. (2018). Reduced-dimensional α-CsPbX3 perovskites for efficient and stable photovoltaics. Joule.

[17] Ma Q., Huang S., Wen X., Green M.A., Ho-Baillie A.W.Y. (2016). Hole Transport Layer Free Inorganic CsPbIBr2 Perovskite Solar Cell by Dual Source Thermal Evaporation. Adv. Energy Mater..

[18] Subhani W.S., Wang K., Du M., Wang X., Liu S. (2019). Interface-Modification-Induced Gradient Energy Band for Highly Efficient CsPbIBr2 Perovskite Solar Cells. Adv. Energy Mater..

[19] Jiang F., Choy W.C., Li X., Zhang D., Cheng J. (2015). Post-treatment-Free Solution-Processed Non-stoichiometric NiOx Nanoparticles for Efficient Hole-Transport Layers of Organic Optoelectronic Devices. Adv. Mater..

[20] Wang X., Deng L.-L., Wang L.-Y., Dai S.-M., Xing Z., Zhan X.-X., Lu X.-Z., Xie S.-Y., Huang R.-B., Zheng L.-S. (2017). Cerium oxide standing out as an electron transport layer for efficient and stable perovskite solar cells processed at low temperature. J. Mater. Chem. A.

[21] Xing Z., Li S.-H., Wu B.-S., Wang X., Wang L.-Y., Wang T., Liu H.-R., Zhang M.-L., Yun D.-Q., Deng L.-L. (2018). Photovoltaic performance and stability of fullerene/cerium oxide double electron transport layer superior to single one in p-i-n perovskite solar cells. J. Power Sources.

[22] Woo K., Kim Y., Moon J. (2012). A non-toxic, solution-processed, earth abundant absorbing layer for thin-film solar cells. Energy Environ. Sci..

[23] Lu J., Chen S.-C., Zheng Q. (2018). Defect Passivation of CsPbIBr2 Perovskites for High-Performance Solar Cells with Large Open-Circuit Voltage of 1. 28 V. ACS Appl. Energy Mater..

[24] Shrotriya V., Li G., Yao Y., Chu C.W., Yang Y. (2006). Transition metal oxides as the buffer layer for polymer photovoltaic cells. Appl. Phys. Lett..

[25] Yang B., Chen H., Shuang X., Xue Q., Teng Z., Zhu Z., Qiang L., Chen H., Yun Y., Hu Z. (2016). Effects of a Molecular Monolayer Modification of NiO Nanocrystal Layer Surfaces on Perovskite Crystallization and Interface Contact toward Faster Hole Extraction and Higher Photovoltaic Performance. Adv. Funct. Mater..

[26] Liu Z., Zhu A., Cai F., Tao L., Zhou Y., Zhao Z., Qi C., Cheng Y.B., Zhou H. (2017). Nickel oxide nanoparticles for efficient hole transport in p-i-n and n-i-p perovskite solar cells. J. Mater. Chem. A.

[27] Dong Y., Xin Z., Yang R., Zhou Y., Wei Y., Wang X., Li C., Liu S.F., Chang R. (2016). Surface Optimization to Eliminate Hysteresis for Record Efficiency Planar Perovskite Solar Cells. Energy Environ. Sci..

[28] Bi C., Wang Q., Shao Y., Yuan Y., Xiao Z., Huang J. (2015). Non-wetting surface-driven high-aspect-ratio crystalline grain growth for efficient hybrid perovskite solar cells. Nat. Commun..

[29] Yun S.-C., Ma S., Kwon H.-C., Kim K., Jang G., Yang H., Moon J. (2019). Amino Acid Salt-Driven Planar Hybrid Perovskite Solar Cells With Enhanced Humidity Stability. Nano Energy.

[30] Zhu W., Shen C., Wu Y., Zhang H., Li E., Zhang W., Xu X., Wu W., Tian H. (2019). Semi-Locked Tetrathienylethene as Promising Building Block for Hole Transporting Materials: Toward Efficient and Stable Perovskite Solar Cells. Angew. Chem. Int. Ed..

[31] Cheng R., Chung C.C., Zhang H., Zhou Z., Zhai P., Huang Y.T., Lee H., Feng S.P. (2019). An Air Knife–Assisted Recrystallization Method for Ambient-Process Planar Perovskite Solar Cells and Its Dim-Light Harvesting. Small.

[32] Yang J., Chen S., Xu J., Zhang Q., Liu H., Liu Z., Yuan M. (2019). A Review on Improving the Quality of Perovskite Films in Perovskite Solar Cells via the Weak Forces Induced by Additives. Appl. Sci..

